# An Image-Based Quantized Compressive Sensing Scheme Using Zadoff–Chu Measurement Matrix

**DOI:** 10.3390/s23021016

**Published:** 2023-01-16

**Authors:** Linlin Xue, Weiwei Qiu, Yue Wang, Zhongpeng Wang

**Affiliations:** School of Information and Electronic Engineering, Zhejiang University of Science and Technology, Hangzhou 310023, China

**Keywords:** block compressive sensing, Zadoff–Chu matrix, quantization

## Abstract

In this paper, a complex-valued Zadoff–Chu measurement matrix is proposed and used in an image-based quantized compressive sensing (CS) scheme. The results of theoretical analysis and simulations show that the reconstruction performance generated by the proposed Zadoff–Chu measurement matrix is better than that obtained by commonly used real-valued measurement matrices. We also applied block compressive sensing (BCS) to reduce the computational complexity of CS and analyzed the effect of block size on the reconstruction performance of the method. The results of simulations revealed that an appropriate choice of block size can not only reduce the computational complexity but also improve the accuracy of reconstruction. Moreover, we studied the effect of quantization on the reconstruction performance of image-based BCS through simulations, and the results showed that analog-to-digital converters with medium resolutions are sufficient to implement quantization and achieve comparable reconstruction performance to that obtained at high resolutions, based on which an image-based BCS framework with low power consumption can thus be developed.

## 1. Introduction

With the development of multimedia technology, communication networks are required to transmit and process a large number of images at any given time. The image signals generally consume a large amount of storage space, and compressive sensing (CS) has been widely used in image transmission to reduce the cost of storage and the requisite channel bandwidth [1,2,3,4]. CS assumes that a sparse or compressible signal can be sampled at sub-Nyquist rates on the transmission side, and the original signal can be reconstructed from these samples with a high probability of success on the receiver side [5]. Various CS schemes have been proposed, but the quality of reconstruction of image-based CS still lags far behind that of conventional compression schemes, such as JPEG and JPEG2000. Obtaining an adequate reconstruction thus remains a pressing issue in CS. The measurement matrix plays an important role in the reconstruction performance of the CS framework. Conventional measurement matrices are usually real valued, such as the chaotic measurement matrix [6], partial Hadamard matrix [7], Toeplitz matrix [8], and Bernoulli matrix [9], and complex-valued measurement matrices have rarely been used. To improve the quality of reconstruction of CS, a complex-valued measurement matrix is introduced in this paper, and block CS (BCS) is used to reduce its computational complexity [10]. Quantization, which should be considered in applications [11,12], is also discussed, and useful conclusions are obtained for the framework of image-based BCS.

### 1.1. Related Work

The quality of reconstruction of sparse signals mainly depends on the design and choice of the measurement matrix. Real-valued measurement matrices have been used in most studies on image-based CS, while complex-valued matrices have received little attention. The authors of Ref. [13] proposed a complex-valued BCS scheme in which a complex-valued partial Hadamard matrix serves as the measurement matrix. The results of experiments showed that the quality of reconstruction obtained by using a complex-valued measurement matrix is better than that obtained by using real-valued measurement matrices. The authors of Ref. [14] proposed an efficient recovery algorithm through the complex-to-real transformation of CS, where a complex-valued discrete Fourier transform (DFT) matrix was used as the sparse basis matrix. However, the introduction of a complex-valued sparse basis matrix or a complex-valued measurement matrix in the above methods causes the measured data to also become complex, thus increasing the computational complexity of CS. The BCS scheme provides an efficient approach to reduce computational complexity. 

In practical image compressed transmission systems, the measured signal should be quantized into a limited number of bits. This is usually implemented by using multi-bit analog-to-digital converters (ADC) [15]. The quantization inevitably introduces noise that degrades the reconstruction performance of image-based BCS. Many researchers have thus studied the effects of quantization on the reconstruction performance of the quantized CS in recent years and proposed robust strategies to accommodate the quantization errors [16,17,18,19,20]. The authors of ref. [16] presented a detailed theoretical analysis of the effects of quantization-induced noise on the reconstruction performance of CS. Reference [19] derived the upper bound of error in the reconstruction of sparse signals. More recently, the authors of ref. [20] proposed a learning-based quantized CS method for the recovery of sparse signals, and their experimental results showed that this method delivers acceptable performance. However, an image-based quantized BCS framework that uses a complex-valued measurement matrix and the effects of the resolution of ADC on reconstruction performance have rarely been researched. 

### 1.2. Motivation and Contributions

Obtaining high-quality reconstruction performance with low complexity is considered to be a challenging problem for the CS framework. CS has been widely used in image compression and processing. It is important to properly design or choose the measurement matrix that significantly affects the performance of image recovery. Generally, natural image signals are sparse in some orthonormal basis, such as discrete wavelet transform (DWT) or discrete cosine transform (DCT). According to CS theory, the mutual coherence between the sparse basis matrix and measurement matrix can be used to evaluate the reconstruction performance of CS. The smaller the mutual coherence, the better the recovery performance of image-based CS. Therefore, designing and constructing a measurement matrix that has low coherence with the orthonormal sparse basis is important work. Zadoff–Chu sequences have an ideal periodic autocorrelation and constant magnitude. These properties are helpful to the design of a measurement matrix with low mutual coherence. Our initial experiment results show that a complex-valued Zadoff–Chu matrix generated from the Zadoff–Chu sequence has low mutual coherence with the conventional DCT sparse basis matrix. Moreover, inspired by Ref. [13], we consider utilizing a complex-valued Zadoff–Chu matrix as the measurement matrix to improve the recovery performance of image-based CS. Therefore, in this paper, we propose an image-based CS framework that uses the complex-valued Zadoff–Chu measurement matrix. It delivers better reconstruction performance than that obtained by using real-valued measurement matrices. We also apply BCS to the framework to reduce its computational complexity.

In practical image-based CS communication systems, ADCs are usually employed to quantize the compressed data to form a bit stream. For an image-based quantized BCS, the use of high-resolution ADCs can reduce the quantization noise and improve its reconstruction performance. However, the high-resolution ADC consumes a large amount of power and requires costly hardware. A quantized BCS that consumes a small amount of power is desirable. We examine the effects of quantization on the reconstruction performance of the image-based BCS to develop a quantized BCS framework with medium-resolution ADCs. 

The main contributions of this work are as follows:

(1) We construct a complex-valued measurement matrix based on a Zadoff–Chu sequence. The results of theoretical analysis and simulations show that it outperforms conventional real-valued matrices in terms of reconstruction performance.
(2) We apply BCS to reduce the computational complexity of the CS framework and analyze the effect of block size on its reconstruction performance. The results of simulations show that an appropriate block size can reduce the computational complexity as well as improve the accuracy of reconstruction of the framework.
(3) We examine the effects of quantization on the reconstruction performance of the proposed image-based BCS. The results of simulations show that an ADC with a medium resolution is sufficient for it to implement quantization and achieve comparable reconstruction performance to that of an ADC with a high resolution. This can be used to develop an image-based quantized BCS framework with low power consumption.

## 2. Principles of Image-Based BCS with Zadoff–Chu Matrix

We now present details of the proposed image-based quantized BCS framework that uses the complexed-valued Zadoff–Chu measurement matrix. A test image is first divided into small non-overlapping blocks and then transformed into sparse signals by using a sparse basis. The resulting sparse signal is compressively sampled by a partial complex-valued Zadoff–Chu measurement matrix that is controlled by keys generated from a chaotic map to ensure the security of the data. Following this, the measured samples are quantized and transmitted through the channel. On the receiver side, the corresponding inverse operations are carried out and a reconstructed image is obtained. 

### 2.1. Image-Based BCS Framework

A test image of size N×N is first divided into non-overlapping image blocks of size $N_{B}\times N_{B}$. The number of image blocks is $(N/N_{B})\times (N/N_{B})$. Each image block is then transformed into a sparse signal by a sparse basis and compressively sampled by a complexed-valued measurement matrix of size $M_{B}\times N_{B}(M_{B}<N_{B})$. 

Let $x_{i}^{j}$ denote the *i*-th column of the *j*-th image block. $x_{i}^{j}$ is transformed into a sparse signal $s_{i}^{j}$ by a sparse matrix $\Psi_{B}$ of size $N_{B}\times N_{B}$. $x_{i}^{j}$ can then be expressed as

(1) $$x_{i}^{j}=\Psi_{B}s_{i}^{j}$$

The measurement process of BCS can then be expressed as

(2) $$y_{i}^{j}=\Phi_{B}\Psi_{B}s_{i}^{j}$$

where $\Phi_{B}$ is the measurement matrix of size $M_{B}\times N_{B}$. $A_{B}=\Phi_{B}\Psi_{B}$ is called the sensing matrix, and it should satisfy the Restricted Isometry Property [21], as expressed in Equation (3), to reconstruct the original image block:

(3) $$1-\varepsilon \leq \frac{{\left\|A_{B}s_{{}_{i}}^{j}\right\|}_{2}}{\left\|s_{i}^{j}\right\|}\leq 1+\varepsilon$$

where ε∈[0, 1]. After all $x_{i}^{j}$ have been measured, the obtained compressed data $y_{i}^{j}$ form a matrix $Y^{j}$ of size $M_{B}\times N_{B}$, which can be expressed as

(4) $$Y^{j}=\left[\begin{array}{cccc} y_{1}^{j} & y_{2}^{j} & \cdots & y_{N_{B}}^{j} \end{array}\right]$$

On the receiver side, the sparse signal can be reconstructed through ${𝓁}_{1}$-minimization or greedy methods by solving the following optimization problem [22]:

(5) $$\underset{s_{i}^{j}}{\min}{\left\|s_{i}^{j}\right\|}_{1}\begin{array}{cc} & s.t.\begin{array}{cc} & y_{i}^{j} \end{array} \end{array}=\Phi_{B}\Psi_{B}s_{i}^{j}$$

Then, according to $x_{i}^{j}=\Psi_{B}^{}s_{i}^{j}$, the reconstructed signal of the *j*-th image block can be obtained and expressed as $x^{j}=\left[\begin{array}{cccc} x_{1}^{j} & x_{2}^{j} & \cdots & x_{N_{B}}^{j} \end{array}\right]$. Finally, all reconstructed image blocks are used to reconstruct the entire original image X. 

A number of reconstruction algorithms can be used to solve the optimization problem in Equation (5). We use the orthogonal matching pursuit (OMP) [23] method here. Moreover, a conventional discrete cosine transform (DCT) of size $N_{B}\times N_{B}$ is used as the sparse basis matrix $\Psi_{B}$, and the complex-valued Zadoff–Chu matrix is used as the measurement matrix $\Phi_{B}$. In the BCS scheme, the size of the measurement matrix is usually smaller than that in the entire image CS. Therefore, a smaller amount of memory is required, making the framework more efficient and practical. 

### 2.2. Design of Zadoff–Chu Measurement Matrix

The Zadoff–Chu matrix is generated from a Zadoff–Chu sequence. A standard Zadoff–Chu sequence of length *L* is defined as follows [24]:

(6) $$p(n)=\left\{\begin{array}{c} \exp\left(-j\frac{\pi un(n+1)}{L}\right),\begin{array}{cc} & \begin{array}{cc} L & odd \end{array} \end{array}\\ \;\exp\left(-j\frac{\pi un^{2}}{L}\right)\begin{array}{ccc} , & & \begin{array}{cc} \begin{array}{cc} & L \end{array} & even \end{array} \end{array} \end{array}\right.$$

where $j=\sqrt{-1}$, *u* is the root index, and 0<u<L. To generate the Zadoff–Chu matrix **P**, the Zadoff–Chu sequence of length $L=N^{2}$ is reshaped into a square matrix, as shown in Equation (7):

(7) $$P=\left[\begin{array}{cccc} p_{0,0} & p_{0,1} & \cdots & p_{0,(N-1)}\\ p_{1,0} & p_{1,1} & \cdots & p_{1,(N-1)}\\ \vdots & \vdots & \ddots & \vdots \\ p_{(N-1),0} & p_{(N-1),1} & \cdots & p_{\left(N-1),(N-1\right)} \end{array}\right]$$

A partial Zadoff–Chu measurement matrix can then be obtained by randomly selecting *M* rows from the matrix P:

(8) Φ=DRP

where $P\in {\mathbb{R}}^{N\times N}$ is the Zadoff–Chu matrix, $R\in {\mathbb{R}}^{N\times N}$ is a row permutation matrix generated from a chaotic logistic map, and RP denotes the multiplication of R by P from the left. $D\in {\mathbb{R}}^{N\times N}$ is a selection matrix that is used to choose *M* rows from the matrix RP. 

The permutation matrix, which has exactly one entry of “1” in each row and each column and “0”s elsewhere, can be expressed as,

(9) $$R=\left[\begin{array}{cccc} 0 & 1 & \cdots & 0\\ 1 & 0 & \cdots & 0\\ \vdots & \vdots & \ddots & \vdots \\ 0 & 0 & \cdots & 1 \end{array}\right]$$

We generate the permutation matrix by a chaotic logistic map, which is defined as follows [25]:

(10) $$z_{i+1}=\mu z_{i}(1-z_{i})$$

where μ∈(3.99996,4] denotes the control parameter and $z_{0}$ is the initial value of the logistic map. In the simulations reported in this paper, the initial value and control parameter of the logistic map were set to $z_{0}=0.33$ and μ=4, respectively.

Based on Equation (10), an integer chaotic sequence $a=\left[\begin{array}{cccc} a(1) & a(2) & \ldots & a(N) \end{array}\right]$ can be obtained according to the following formula:

(11) $$a_{n}=\mathrm{mod}(floor(z_{n}+100)\times 10^{10}),N)+1$$

where floor(x) returns the nearest integer less than or equal to x, and $\mathrm{mod}(x,N_{B})$ returns the remainder of x when divided by *N_B_*. Then, the permutation matrix $R_{chaos}$ based on the chaotic sequence **a** can be expressed as,

(12) $$R_{chaos}=\left[\begin{array}{cccc} 0 & R_{1,a(1)} & \cdots & 0\\ R_{2,a(2)} & 0 & \cdots & 0\\ \vdots & \vdots & \ddots & \vdots \\ 0 & 0 & \cdots & R_{N,a(N)} \end{array}\right]$$

In Equation (12), $R_{chaos}$ has exactly one entry of “1” in each row and each column, and “0”s elsewhere.

We update the matrix RP by $R_{chaos}P$, and the partial Zadoff–Chu measurement matrix expressed by Equation (8) can then be rewritten as,

(13) $$\Phi =DR_{chaos}P$$

Finally, the measurement matrix Φ of size M×N is obtained. The measurement matrix of the BCS scheme is denoted by $\Phi_{B}$ and its size is adjusted to $M_{B}\times N_{B}$.

### 2.3. Image-Based Quantized BCS Framework

**Figure 1:**
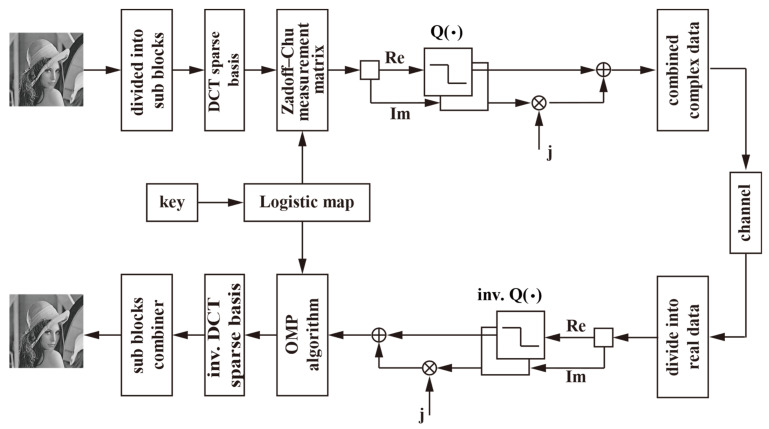
The proposed image-based quantized BCS framework.

Figure 1 shows the proposed image-based quantized BCS framework based on the complex-valued Zadoff–Chu measurement matrix. Its main steps can be summarized as follows:

Step 1: Image block partitioning. The original image of size N×N is divided into non-overlapping blocks of size $N_{B}\times N_{B}$. 

Step 2: Sparse transformation. The image block of size $N_{B}\times N_{B}$ is transformed to a new domain by using the DCT sparse basis $\Psi_{B}$.

Step 3: Compressed sampling. The transformed image data are sampled by the complex-valued Zadoff–Chu measurement matrix $\Phi_{B}$ of size $M_{B}\times N_{B}$. The measurement matrix is constructed according to Equation (13). 

Step 4: Quantization. The real and imaginary parts of the measured data of the image block are quantized according to the following formula:

(14) $$P_{3}=round((2^{B}-1)\times (P_{1}-\min(P_{1}))/(\max(P_{1})-\min(P_{1})))$$

(15) $$P_{4}=round((2^{B}-1)\times (P_{2}-\min(P_{2}))/(\max(P_{2})-\min(P_{2})))$$

where *B* denotes the number of bits representing each measurement, and $P_{1}$ and $P_{2}$ denote the real part and the imaginary part of the measured image block matrix, respectively. max(⋅) and min(⋅) denote the maximum and the minimum values of the matrix, respectively. The quantized data are then transmitted through the channel. 

Step 5: Inverse quantization. On the receiver side, the quantized data are inversely quantized according to the following:

(16) $${\hat{P}}_{1}=P_{3}\times \frac{\max(P_{1})-\min(P_{1})}{2^{B}-1}+\min(P_{1})$$

(17) $${\hat{P}}_{2}=P_{4}\times \frac{\max(P_{2})-\min(P_{2})}{2^{B}-1}+\min(P_{2})$$

Step 6: Recovering the sparse signal for each complex-valued image block. In this step, the conventional OMP algorithm is applied. 

Step 7: Inverse sparse transformation. Each recovered spare signal is converted into the original image block by an inverse DCT sparse transformation. Finally, all image blocks are combined to form the entire reconstructed image.

## 3. Performance of Measurement Matrices

The reconstruction performance of CS depends on designing and choosing an appropriate measurement matrix. A perfect CS requires that the measurement matrix and the sparse matrix be incoherent. In the subsequent analysis, we use the average mutual coherence of the sensing matrix **A** to measure the coherence between the measurement matrix and the sparse matrix. This is defined as [26].

(18) $$\mu_{av}=\frac{1}{N(N-1)}\sum_{i=1}^{N}\sum_{\begin{array}{l} j=1\\ j\neq i \end{array}}^{N}\frac{\left|a_{i}^{T}a_{j}\right|}{{\left\|a_{i}\right\|}_{2}{\left\|a_{j}\right\|}_{2}}$$

where **a***_i_* and **a***_j_* represent the *i*-th and the *j*-th column of sensing matrix **A**, respectively. In general, the smaller the average mutual coherence, the better the reconstruction performance of CS.

The average mutual coherence of sensing matrix **A** is calculated according to Equation (18). Table 1 shows the results for different measurement matrices under different compressive ratios (CRs) and block sizes. In our experiments, the conventional DCT matrix served as the sparse basis matrix and five measurement matrices—namely, the partial Zadoff–Chu measurement matrix, partial complex-valued Hadamard matrix, partial real-valued Hadamard matrix, Toeplitz matrix, and Bernoulli matrix—were used as the measurement matrices in turn. 

Table 1 shows that the average mutual coherence of the complex-valued Zadoff–Chu measurement matrix was lower than those of the other measurement matrices in almost all cases. Based on the results of Table 1, we can conclude that the reconstruction performance of the proposed scheme was better than that of schemes using the other measurement matrices. This deduction was confirmed by simulation results in the following experiments. In addition, the mutual coherence of all measurement matrices decreased with an increase in the CR. This was apparent as higher CR corresponded to better reconstruction performance.

## 4. Simulations and Analysis 

In this section, simulations of the proposed quantized BCS scheme by using MATLAB 2019b installed on a laptop with a 1.8 GHz i7-8550U CPU and 16 GB RAM. “Lena”, “Cameraman”, “Boats”, “Foreman”, and “House” were used as the test images. The reconstruction performance of different measurement matrices was compared and the effect of block size on it was analyzed.

We used structural similarity (SSIM) and the peak signal-to-noise (PSNR) to qualitatively evaluate the reconstruction performance of the proposed BCS scheme. The SSIM is defined as [27],

(19) $$SSIM\left(x,y\right)=\frac{\left(2\mu_{x}^{}\mu_{y}^{}+C_{1})(\sigma_{xy}+C_{2}\right)}{\left(\mu_{x}^{2}+\mu_{y}^{2}+C_{1})(\sigma_{x}^{2}+\sigma_{y}^{2}+C_{2}\right)}$$

where x and y are the original image and the reconstructed image, respectively, $\sigma_{x}^{2}$ and $\sigma_{y}^{2}$, and $\;\mu_{x}$ and $\mu_{y}$ denote the variances and the means of x and y, respectively, $\sigma_{xy}$ denotes the covariance of *x* and *y*, and $C_{1}$ and $C_{2}$ are positive constants used to prevent a null denominator. In general, a higher SSIM indicates better similarity between images. 

The PSNR is defined as [28,29],

(20) $$\mathrm{PSNR}=10\cdot \log_{10}\left(\frac{255^{2}}{\mathrm{MSE}}\right)$$

where the mean-squared error (MSE) is defined as,

(21) $$\mathrm{MSE}=\frac{1}{M_{1}\times N_{1}}\sum_{i=0}^{M_{1}-1}\sum_{j=0}^{N_{1}-1}{\left(X(i,j)-Y(i,j)\right)}^{2}$$

where $M_{1}$ and $N_{1}$ are the numbers of pixels along the horizontal and the vertical coordinates of the image, respectively, and *X*(*i*,*j*) and *Y*(*i*,*j*) represent the original image and the reconstructed image, respectively. A higher PSNR indicates better reconstruction performance.

### 4.1. Comparison of Reconstruction Performance

In this section, we calculate the SSIM and PSNR for different measurement matrices based on the OMP reconstruction algorithm, as shown in Table 2. The “Lena” image of size 256 × 256 was used as the test image. It is clear from the table that the reconstruction performance of the complex-valued Zadoff–Chu matrix and the complex-valued Hadamard matrix was better than that of the other real-valued measurement matrices—namely the real-valued Hadamard matrix, Toeplitz matrix, and Bernoulli matrix. For instance, when the block size was 256 × 256 and CR was 0.6, the PSNRs of the complex-valued Zadoff–Chu matrix, complex-valued Hadamard matrix, real-valued Hadamard matrix, Toeplitz matrix, and Bernoulli matrix were 29.3149 dB, 29.2307 dB, 27.1256 dB, 25.9744 dB, and 25.5922 dB, respectively. These results coincide with those inferred based on mutual coherence shown in Table 1. In addition, the reconstruction performance of the matrices for block sizes of 256 × 256, 128 × 128, and 64 × 64 was better than that for image blocks of sizes 32 × 32 and 16 × 16. Furthermore, the differences of reconstruction performance among block sizes of 256 × 256, 128 × 128, and 64 × 64 were minor.

**Figure 2:**
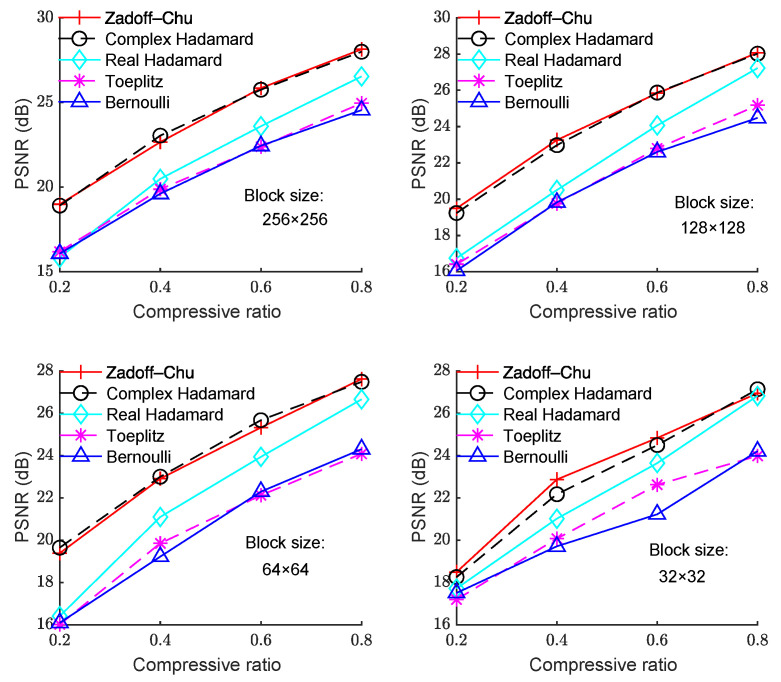
PSNR with CR under different block sizes for the “Cameraman” image.

**Figure 3:**
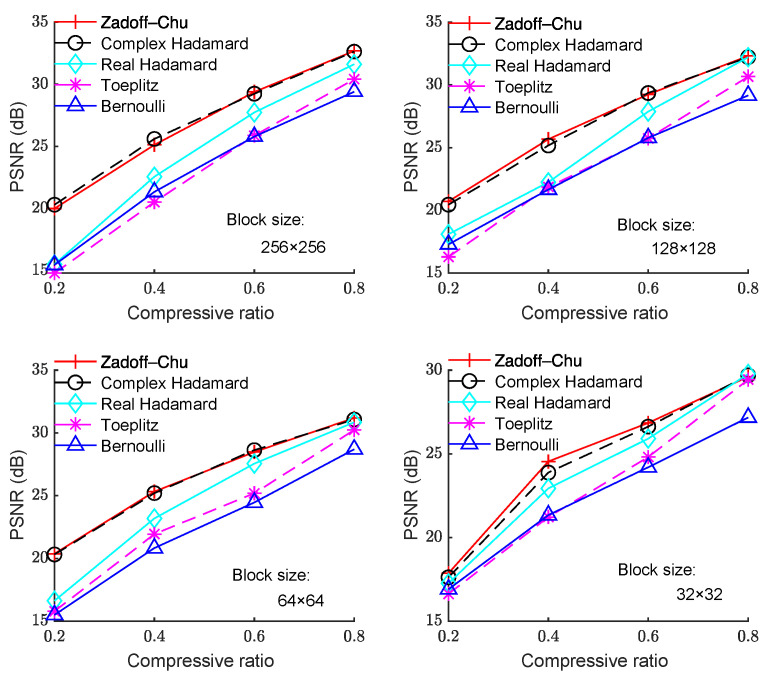
PSNR with CR under different block sizes for the “Boats” image.

**Figure 4:**
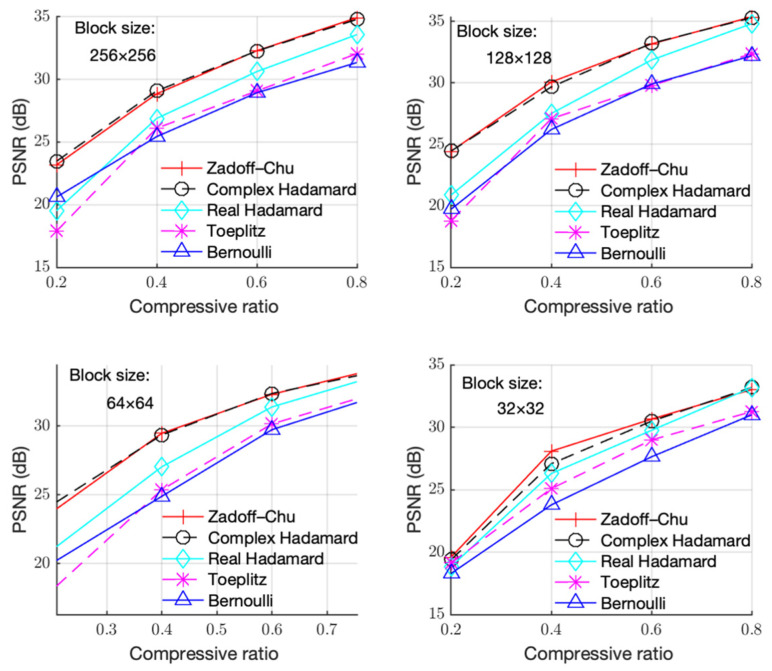
PSNR with CR under different block sizes for the “Foreman” image.

**Figure 5:**
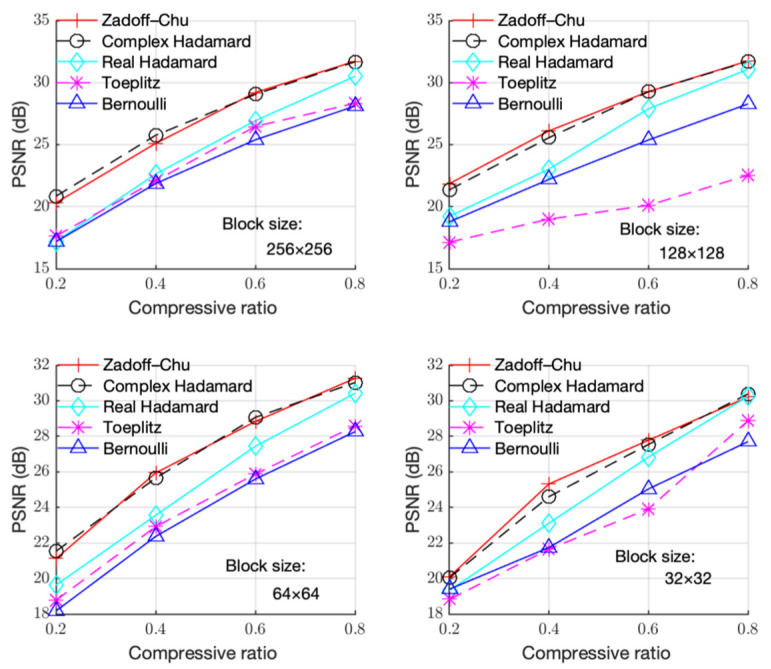
PSNR with CR under different block sizes for the “House” image.

We also ran simulations on the other test images, and the results are shown in Figure 2, Figure 3, Figure 4 and Figure 5. Figure 2 shows the PSNR with CR for different measurement matrices on the “Cameraman” image. In Figure 2, the block sizes are 256 × 256, 128 × 128, 64 × 64, and 32 × 32 from left to right, and from top to bottom. It can be seen that the PSNR performance using the Zadoff–Chu matrix is much better than that using the real-valued Hadamard matrix, Toeplitz matrix, and Bernoulli matrix for different block sizes, while the PSNR performance for the Zadoff–Chu matrix and complex Hadamard matrix are similar. In addition, the PSNR performance for block size of 32 × 32 degraded compared with that for 256 × 256, 128 × 128, and 64 × 64. Moreover, the difference of PSNR performance for block size of 256 × 256, 128 × 128, and 64 × 64 was minor.

Figure 3, Figure 4 and Figure 5 show the PSNR performance using different measurement matrices under different block sizes for the “Boats”, “Foreman”, and “House” images, respectively. Based on Figure 3, Figure 4 and Figure 5, similar conclusion can be drawn as that of Figure 2. Hence, the Zadoff–Chu measurement matrix provided high reconstruction performance and it was effective for different images. 

In the above experiments, the reconstruction performance of the proposed image-based BCS was evaluated using the OMP reconstruction algorithm. In this subsection, the SL0 reconstruction algorithm [30] is used to study the reconstruction performance of the image-based BCS with different measurement matrices, and the calculated results are shown in Table 3. The “Lena” image of size 256 × 256 is also served as the test image. The PSNR and mean absolute error (MAE) [31] are used to evaluate the reconstruction performance. Generally, the smaller the MAE value, the better the reconstruction performance of BCS scheme, which is opposite to that of PSNR. It is clear from Table 3 that the reconstructed performance of BCS scheme using the Zadoff–Chu measurement matrix is much better than that using the other real-valued measurement matrices under different CRs and different block sizes in terms of PSNR and MAE metrics. Even compared with the complex-valued Hadamard matrix, the proposed Zadoff–Chu matrix also has obvious advantage in reconstruction performance. These conclusions drawn from Table 3 are similar to those obtained from Table 2, further showing the effectiveness of the proposed Zadoff–Chu matrix.

### 4.2. Analysis of Computational Complexity 

**Figure 6:**
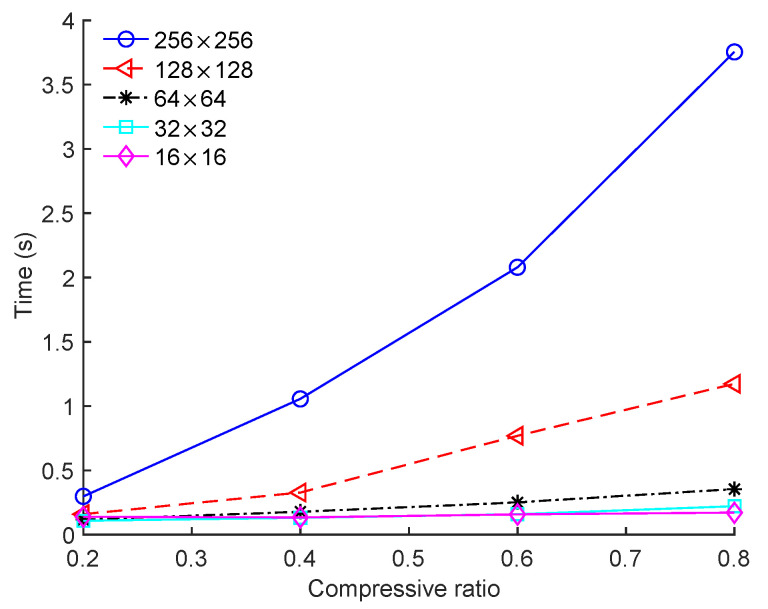
Run times of image-based BCS under different block sizes on the “House” image.

Figure 6 shows the run time of the image-based BCS using the proposed Zadoff–Chu measurement matrix for different block sizes, where the “House” image was used as the test image. The run time of the image-based BCS decreased significantly when the block size was reduced from 256 × 256 to 64 × 64. However, when the block size was reduced to 32 × 32 and 16 × 16, the run time continued to decrease but at a much lower rate. For instance, when the CR was 0.6, the run times were 2.0791 s, 0.7694 s, 0.2509 s, 0.1588 s, and 0.1569 s for block sizes 256 × 256, 128 × 128, 64 × 64, 32 × 32, and 16 × 16, respectively. 

**Figure 7:**
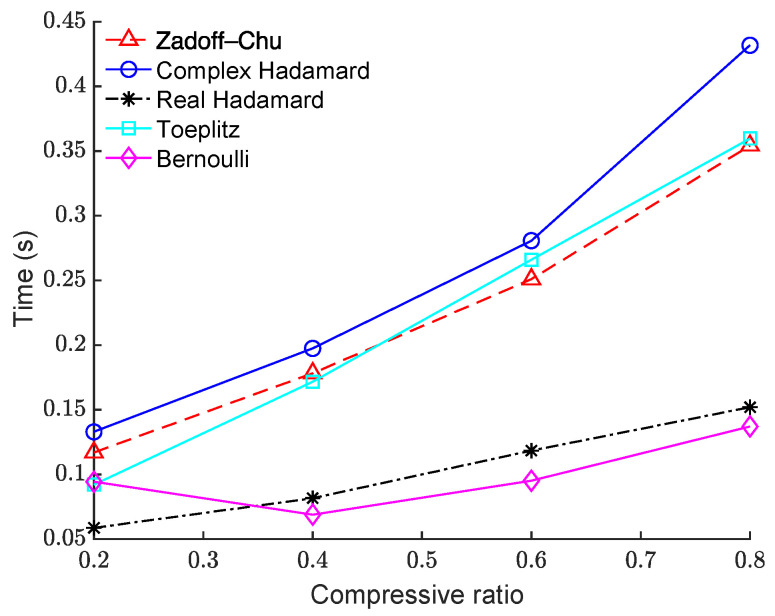
Comparison of the run times of different measurement matrices on the “House” image.

We also compared the run times of the proposed image-based BCS using the Zadoff–Chu matrix with those using the other measurement matrices, as shown in Figure 7. The “House” image was used as the test image and image block size was 64 × 64. The computational complexity of the proposed scheme was higher than schemes that used real-valued matrices, such as the real Hadamard matrix and the Bernoulli matrix. However, its reconstruction performance was significantly better, as shown in Figure 2, Figure 3, Figure 4 and Figure 5. In addition, its computational complexity was lower than that of the complex Hadamard matrix even though their reconstruction performance was similar. Therefore, the proposed Zadoff–Chu matrix is preferable as the measurement matrix on the whole. 

### 4.3. Quantization Error

The quantization of the measured signal inevitably introduces errors to the CS framework. In Ref. [32], the authors analyzed the effects of quantization on the reconstruction of 1D signals based on *B*-bit quantized measurements. In this subsection, we evaluate the effects of *B*-bit quantization on the accuracy of reconstruction of image-based BCS using a complex-valued measurement matrix. 

The quantization implemented by an ADC device is a very important part of the quantized BCS scheme. The number of bits representing each measurement is usually defined as the resolution of an ADC. For instance, if a measured value is denoted by *B* bits, the ADC has a *B*-bit resolution. In general, the higher the resolution of ADC, the smaller the quantization error. 

For an image-based CS scheme, the quantization of the measured complex-valued image data can be simply expressed as,

(22) $$\begin{array}{c} \hat{Y}=\Phi X+e\\ \;\;\;\;\;\;\;\;\;\;\;\;\;\;\;\;\;\;\;\;\;\;\;\;\;\;\;\;\;\;\;\;\;\;\;\;\;\;\;\;\;\;\;\;\;\;\;\;\;\;\;\;\;\;\;\;\;\;\;\;\;\;\;\;=digital_{B}\left\{ℜ\left\{\Phi X\right\}\right\}+jdigital_{B}\left\{I\left\{\Phi X\right\}\right\} \end{array}$$

where ΦX and $\hat{Y}$ denote the measured image data and the quantized measured image data, respectively. In general, the quantized image data are different from the original image data due to truncation or rounding errors, and the difference between them is called the quantization error (noise). It is denoted by **e** in Equation (22). In addition, the subscript *B* denotes the resolution of the ADC used for quantization. 

For real-valued quantization, the error **e** can be represented as an additive noise signal [33]. Hence, for a *B*-bit quantizer with a quantization step of Δ, the mean value of **e** is zero and its variance is,

(23) $$\sigma_{e\_r}^{2}=\Delta^{2}/12$$

where $\Delta {\;=\;2}^{-B}$.

However, for complex-valued quantization, both the real and the imaginary parts of the quantization error contribute to noise. In this case, the variance of the quantization noise (or average power of quantization noise) is given by,

(24) $$\sigma_{e}^{2}=2\Delta^{2}/12=\Delta^{2}/6$$

Furthermore, for a *B*-bit quantizer, the signal-to-quantization noise ratio (SQNR) is defined as [33]

(25) $$\mathrm{SQNR}=10\log_{10}\frac{\sigma_{x}^{2}}{\sigma_{e}^{2}}\;\mathrm{dB}$$

where $\sigma_{x}^{2}$ denotes the average power of the complex-valued measurements. From Equation (25), it is clear that each additional bit in the ADC increases the SQNR due to a reduction in the step size Δ. The level of quantization-induced noise depends on the resolution of the ADC. 

**Figure 8:**
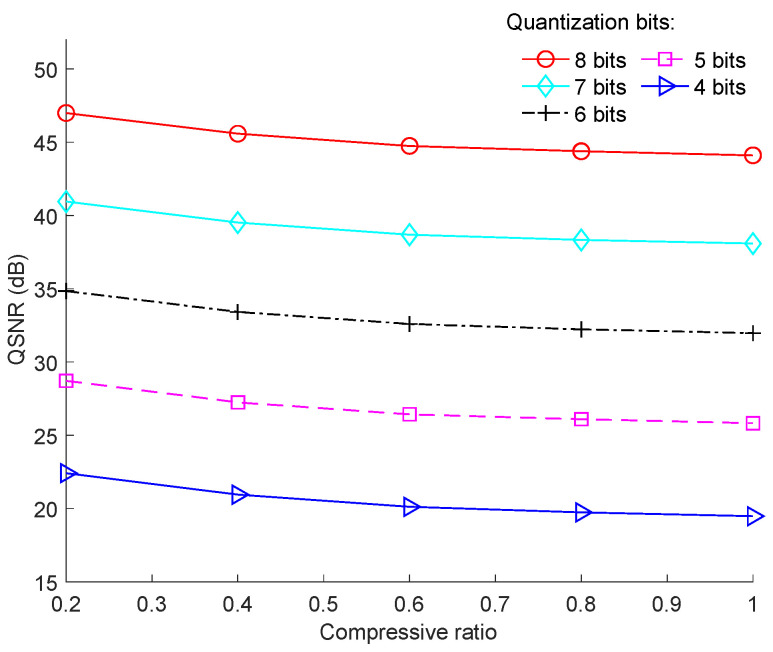
Effects of the resolution of ADC on QSNR for the “Cameraman” image with a block size of 128 × 128.

The effect of the resolution of ADC on QSNR was evaluated and the results are shown in Figure 8, where “Cameraman” was used as the test image. It is clear that the higher the resolution of ADC was, the better was the performance in terms of QSNR. Specifically, each additional bit in the ADC increased the QSNR by approximately 6 dB. 

**Figure 9:**
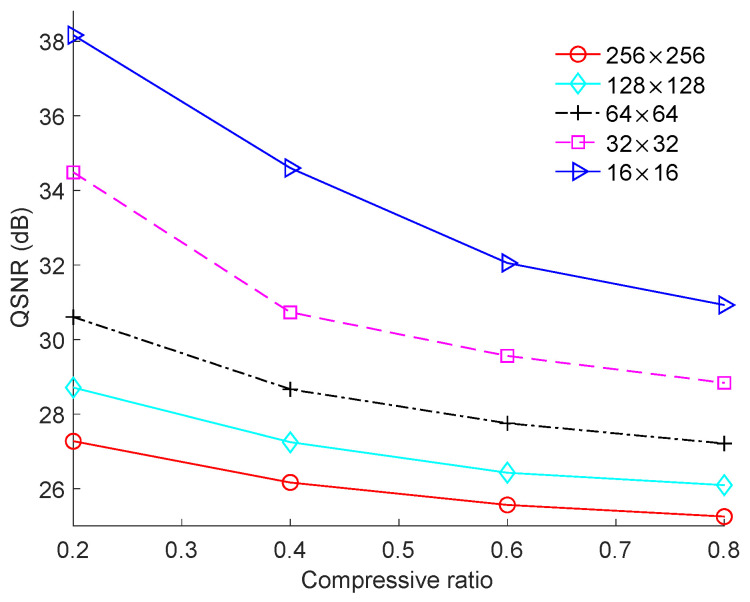
Effect of block size on QSNR performance for the “Cameraman” image under a 5-bit resolution of ADC.

We also simulated the effect of block size on the QSNR performance, as shown in Figure 9. The “Cameraman” image was again used as the test image and the resolution of the ADC was fixed to 5-bit. The QSNR increased with the decrease in block size because quantization was implemented in blocks. The smaller the block size was, the smaller was the quantization error. The tendency of curves of the QSNR in case of different block sizes for other resolutions of the ADC were similar to that of the 5-bit ADC. 

### 4.4. Effect of Quantization on Reconstruction Performance

The quantization of image-based BCS can be implemented by using an ADC. The higher the resolution of the ADC, the smaller the quantization error. However, the power consumption of the ADC is also related to its resolution, where a higher resolution requires more power. Hence, the optimal resolution of the ADC needs to be determined. 

In the image-based BCS framework, the reconstruction algorithm generates errors. When quantization is performed, the quantization error is added to this reconstruction error, making the PSNR performance worse. However, if the error caused by the reconstruction algorithm is dominant over the quantization error, a low-resolution ADC can be used to reduce power consumption.

We assessed the effect of quantization on the reconstruction performance of the proposed image-based quantized BCS scheme that uses the Zadoff–Chu measurement matrix. To explicitly show the degradation of PSNR caused by quantization, we defined PSNR loss (ΔPSNR) as follows:

(26) $$\Delta {\mathrm{PSNR}=\mathrm{PSNR}}_{0}-{\mathrm{PSNR}}_{B-bit}$$

where PSNR_0_ denotes the reconstructed PSNR values without quantization and ${\mathrm{PSNR}}_{B-bit}$ denotes the reconstructed PSNR values when quantization was performed by using an ADC with a *B*-bit resolution. 

**Figure 10:**
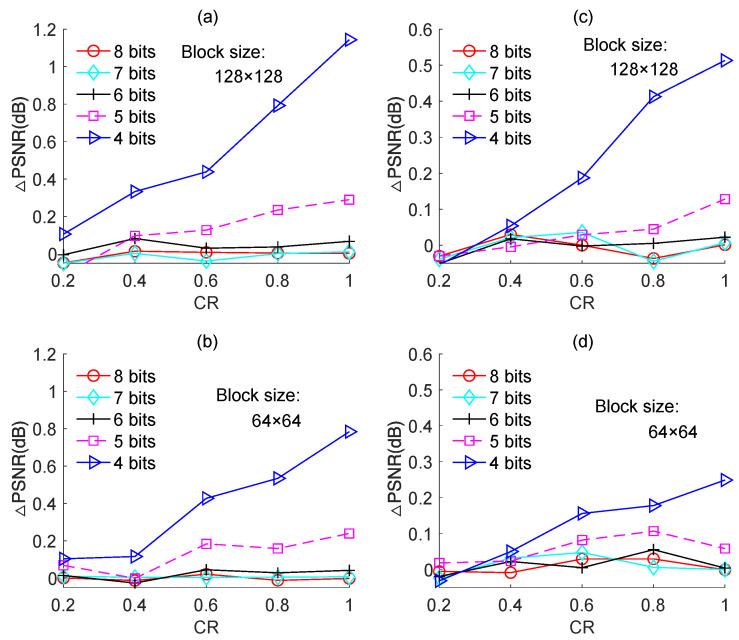
PSNR losses of the quantized BCS with different resolutions of ADC for the “Cameraman” image. (**a**,**b**) are based on Zadoff–Chu measurement matrix; (**c**,**d**) are based on real-valued Hadamard measurement matrix.

Figure 10 shows the PSNR loss in response to the CR for an ADC with different resolutions. Two measurement matrices were used in the simulations, Figure 10a,b correspond to the Zadoff–Chu measurement matrix, and Figure 10c,d correspond to the real-valued Hadamard measurement matrix. The “Cameraman” image of size 256 × 256 was used as the test image. The experiment was performed only for block sizes of 128 × 128 and 64 × 64, because the two block sizes are optimal for image-based BCS in terms of reconstruction performance and computational complexity.

For the case of using the Zadoff–Chu measurement matrix, it is clear from Figure 10a,b that for ADCs with 8-bit, 7-bit, and 6-bit resolutions, the PSNR loss was negligibly small and the difference in it among the three cases was minor. This means that using an ADC with a 6-bit or 7-bit resolution can yield similar PSNR performance compared to that using an ADC with an 8-bit resolution. When the resolution of the ADC was reduced to 5-bit and 4-bit, the PSNR loss increased, and the higher the CR, the larger the PSNR loss. This can be explained as follows: with the increase in CR, the reconstruction error decreased such that the impact of the quantization error on reconstruction performance increased. As seen in Figure 10a, when the CR was 0.6, the ADC with a 4-bit resolution caused a PSNR loss of ~0.5 dB, but when CR was increased to 0.8 and 1.0, the corresponding PSNR losses increased to 0.8 dB and 1.1 dB, respectively. Therefore, in light of both PSNR performance and power consumption, an ADC with a 4-bit resolution is suitable for image-based BCS with a low CR, but an ADC with a 5-bit resolution is required in case of a higher CR.

For the case of using the real-valued Hadamard measurement matrix, the simulated results were shown in Figure 10c,d, from which similar conclusions can be drawn to that of using the complex-valued Zadoff–Chu measurement matrix. Nevertheless, in this case, the PSNR loss caused by quantization was smaller than that of using the Zadoff–Chu measurement matrix. For instance, when the CR was 0.6 and block size was 128 × 128, the ADC with 4-bit resolution caused a PSNR loss of ~1.2 dB for the case of using the Zadoff–Chu measurement matrix, while caused a PSNR loss of ~0.45 dB for the case of using the Hadamard matrix. The main reason is that, for the case of using the complex-valued Zadoff–Chu measurement matrix, the measured data are also complex-valued; therefore, its quantization error consists of two parts, the real part quantization error and the imaginary part quantization error. However, the quantization error of using the real-valued Hadamard measurement matrix only contains the real part. Consequently, the quantization error of using the real-valued Hadamard measurement matrix is evidently smaller than that of using the complex-valued Zadoff–Chu measurement matrix. As a result, the PSNR loss caused by quantization for the case of using the real-valued Hadamard matrix is smaller when compared to that of using the Zadoff–Chu matrix. 

Figure 10 also shows that the PSNR loss for block size 64 × 64 was smaller than that for 128 × 128. This is because the quantization error for block size 64 × 64 was smaller than that for 128 × 128, as shown in Figure 9. From this aspect, the block size of 64 × 64 is preferable than 128 × 128 for image-based quantized BCS. 

**Figure 11:**
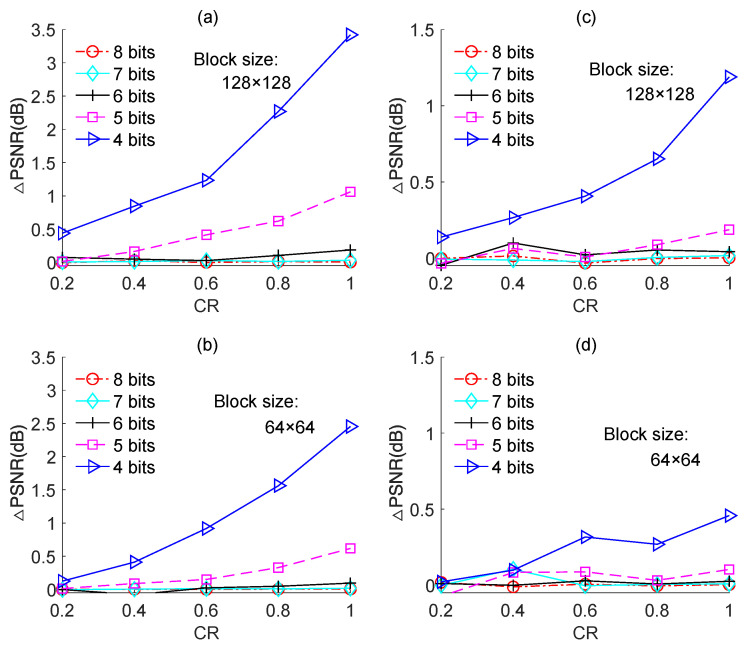
PSNR losses of the quantized BCS with different resolutions of ADC for the “Boats” image. (**a**,**b**) are based on Zadoff–Chu measurement matrix; (**c**,**d**) are based on real-valued Hadamard measurement matrix.

**Figure 12:**
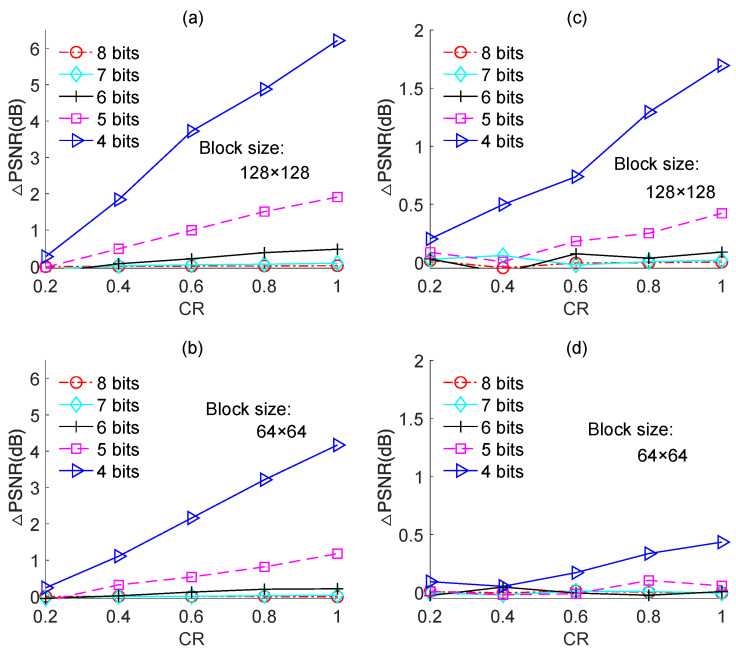
PSNR losses of the quantized BCS with different resolutions of ADC for the “Foreman” image. (**a**,**b**) are based on Zadoff–Chu measurement matrix; (**c**,**d**) are based on real-valued Hadamard measurement matrix.

**Figure 13:**
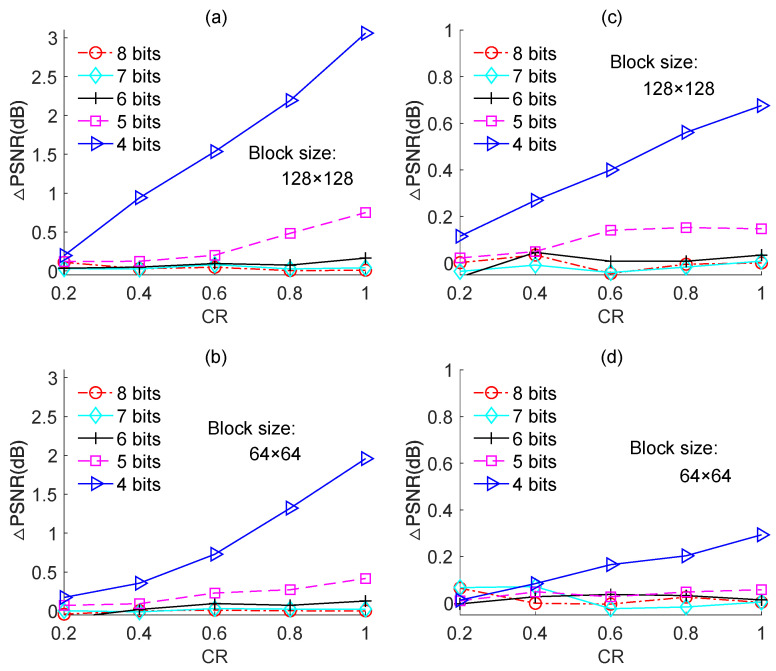
PSNR losses of the quantized BCS with different resolutions of ADC for the “House” image. (**a**,**b**) are based on Zadoff–Chu measurement matrix; (**c**,**d**) are based on real-valued Hadamard measurement matrix.

**Figure 14:**
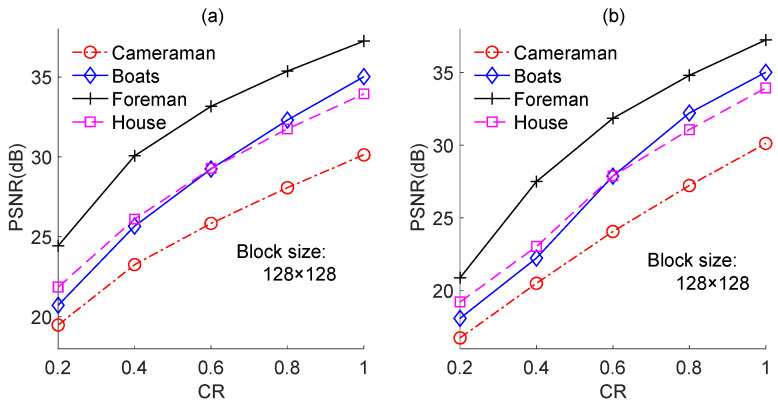
PSNRs of the proposed image-based BCS on different test images. (**a**) is based on Zadoff–Chu measurement matrix; (**b**) is based on real-valued Hadamard measurement matrix.

We also performed experiments on the other test images, namely, “Boats”, “Foreman”, and “House”, and the results are shown in Figure 11, Figure 12 and Figure 13, respectively. The results for each image yielded the same conclusion as was obtained from the results for the “Cameraman” image. However, the PSNR loss caused by quantization was different for different test images. For instance, for image-based BCS using the Zadoff–Chu measurement matrix under a block size of 128 × 128 and a CR of 0.8, the PSNR losses were 0.8 dB, 2.3 dB, 4.9 dB, and 2.2 dB for “Cameraman”, “Boats”, “Foreman”, and “House”, respectively. The PSNR loss resulted from quantization for a test image is related to its PSNR performance. Better PSNR performance means a smaller reconstruction error. In this case, the impact of the quantization error on PSNR performance became comparatively dominant. The results in Figure 14 further support this claim. Figure 14a,b show the PSNRs of the proposed image-based BCS using the complex-valued Zadoff–Chu measurement matrix and real-valued Hadamard matrix, respectively. The block size was 128 × 128 and four images were used as the test images. From Figure 14a, it is clear that when the CR was 0.8, the PSNRs were 27.08 dB, 32.30 dB, 35.36 dB, and 31.85 dB for “Cameraman”, “Boats”, “Foreman”, and “House”, respectively. The better was the PSNR performance, the higher was the PSNR loss caused by quantization. From Figure 14b, a similar conclusion can be draw to that from Figure 14a.

Based on the above analysis, we can conclude the following: (1) The reconstruction error had a dominant impact on the PSNR performance of the proposed quantized BCS framework compared with the influence of quantization error when a high-resolution (8-bit, 7-bit, or 6-bit) ADC was applied. Hence, an ADC with a 6-bit resolution is sufficient to implement quantization and introduces only a slight PSNR loss. (2) At a lower CR, the reconstruction error increases and its impact on PSNR performance becomes more dominant compared with that of the quantization error. In this case, an ADC with a low resolution can be used, such as one with a 5-bit or even a 4-bit resolution.

The benefit of using low resolution ADC is saving power consumption. In practical applications, the optimal resolution of ADC used in image BCS can be determined according to the analysis provided in this paper.

## 5. Conclusions

We proposed a complex-valued Zadoff–Chu measurement matrix here and used it in the image-based quantized BCS framework. The results of theoretical analysis and simulations showed that the complex-valued Zadoff–Chu matrix outperforms conventional real-valued matrices in terms of image reconstruction. We examined its reconstruction performance and computational complexity for different block sizes, based on which optimal block size of image BCS can be determined. We also analyzed the effect of quantization on the proposed image-based BCS. The QSNR and PSNR losses due to quantization were evaluated. The results of simulations showed that an ADC with a medium resolution is sufficient for quantization and yields good reconstruction performance.

## Figures and Tables

**Table 1:** Average mutual coherence of sensing matrix **A** for different measurement matrices. The bold indicates the smallest number (best one) of each row.

Block Size	CR	Partial Zadoff–Chu	Partial Complex-Valued	Partial Real-Valued	Toeplitz Matrix	Bernoulli Matrix
		Matrix (Our Scheme)	Hadamard Matrix [13]	Hadamard Matrix [7]	In [8]	In [9]
256 × 256	0.2	**0.0635**	0.0831	0.0828	0.0995	0.1063
	0.4	**0.0331**	0.0531	0.0569	0.0678	0.0786
	0.6	**0.0177**	0.0335	0.0438	0.0485	0.0639
	0.8	**0.0095**	0.0195	0.0340	0.0344	0.0553
128 × 128	0.2	**0.0938**	0.1306	0.1289	0.1412	0.1502
	0.4	**0.0510**	0.0797	0.0851	0.0975	0.1105
	0.6	**0.0313**	0.0508	0.0634	0.0706	0.0898
	0.8	**0.0167**	0.0290	0.0505	0.0510	0.0780
64 × 64	0.2	**0.1637**	0.1936	0.2028	0.2027	0.2109
	0.4	**0.0895**	0.1206	0.1388	0.1381	0.1535
	0.6	**0.0521**	0.0777	0.1040	0.1035	0.1263
	0.8	**0.0273**	0.0406	0.0769	0.0766	0.1088
32 × 32	0.2	0.2987	0.3042	**0.2891**	0.2892	0.2995
	0.4	**0.1567**	0.1788	0.1953	0.1962	0.2136
	0.6	**0.0867**	0.1125	0.1503	0.1501	0.1751
	0.8	**0.0455**	0.0616	0.1143	0.1142	0.1491
16 × 16	0.2	**0.3463**	0.3799	0.3780	0.3783	0.3852
	0.4	**0.2367**	0.2623	0.2902	0.2886	0.3100
	0.6	**0.1148**	0.1401	0.2011	0.2029	0.2345
	0.8	**0.0679**	0.0764	0.1628	0.1647	0.2014

**Table 2:** Comparison of reconstruction performance between different measurement matrices for “Lena” image based on OMP reconstruction algorithm. The bold indicates the best PSNR value and SSIM value of each row.

Block Size	CR	Zadoff–Chu Matrix		Complex Hadamard		Real Hadamard		Toeplitz		Bernoulli	
		(Our Scheme)		Matrix in [13]		Matrix in [7]		Matrix in [8]		Matrix in [9]	
		PSNR (dB)	SSIM	PSNR (dB)	SSIM	PSNR (dB)	SSIM	PSNR (dB)	SSIM	PSNR (dB)	SSIM
256 × 256	0.2	21.4729	0.8961	**21.7178**	**0.901**	18.2952	0.7875	18.1086	0.7822	18.4498	0.7978
	0.4	25.8956	0.963	**26.0531**	**0.9642**	23.5468	0.9373	22.5806	0.9219	22.3629	0.9181
	0.6	**29.3149**	**0.9833**	29.2307	0.9829	27.1256	0.9725	25.9744	0.9641	25.5922	0.961
	0.8	**31.9906**	**0.991**	31.8765	0.9908	30.3874	0.987	28.7671	0.9812	28.1551	0.9783
128 × 128	0.2	21.8151	0.902	**21.9059**	**0.9051**	19.3419	0.8356	18.328	0.7894	18.9155	0.8189
	0.4	**26.0156**	**0.9638**	25.7342	0.9615	22.9897	0.9291	22.3794	0.9176	21.8435	0.907
	0.6	29.0475	0.9822	**29.1152**	**0.9825**	27.2894	0.9735	25.8591	0.9632	25.5869	0.9608
	0.8	**31.5639**	**0.9901**	31.4874	0.9899	30.9021	0.9884	28.3541	0.9793	28.1683	0.9784
64 × 64	0.2	21.8576	0.9015	**22.0131**	**0.9053**	18.3449	0.7887	18.0126	0.7696	18.129	0.773
	0.4	**25.9802**	**0.9635**	25.8222	0.9622	23.6725	0.9387	22.4263	0.9182	22.5498	0.9208
	0.6	28.4197	0.9794	**28.6781**	**0.9805**	27.1333	0.9724	25.3866	0.9587	24.7357	0.9522
	0.8	**30.895**	**0.9884**	30.7236	0.9879	29.9556	0.9856	28.1744	0.9783	27.7545	0.9762
32 × 32	0.2	**19.9996**	**0.8436**	19.5274	0.8299	19.2267	0.8226	18.7532	0.794	19.2439	0.8133
	0.4	**25.5436**	**0.9594**	24.7436	0.9516	23.7445	0.9399	22.0184	0.9089	21.9432	0.9087
	0.6	**27.6229**	**0.975**	27.3485	0.9735	26.5037	0.9679	24.847	0.953	24.0014	0.943
	0.8	29.8905	0.9853	**30.0824**	**0.986**	29.8765	0.9853	28.0976	0.9779	27.0891	0.9722
16 × 16	0.2	15.5284	0.6342	**22.3741**	**0.9134**	19.1813	0.8321	21.0813	0.8849	21.1405	0.8873
	0.4	**22.5201**	**0.9158**	22.3248	0.9123	21.3433	0.8946	21.1848	0.8863	21.4778	0.8943
	0.6	**26.5552**	**0.9683**	26.5286	0.9681	25.6645	0.9614	24.7753	0.9519	24.5238	0.9494
	0.8	**29.6106**	**0.9843**	29.565	0.9842	29.181	0.9827	26.9895	0.9707	26.0026	0.964

**Table 3:** Comparison of reconstruction performance between different measurement matrices for “Lena” image based on SL0 reconstruction algorithm. The bold indicates the best PSNR value and MAE value of each row.

Block Size	CR	Zadoff–Chu Matrix		Complex Hadamard		Real Hadamard		Toeplitz		Bernoulli	
		(Our Scheme)		Matrix in [13]		Matrix in [9]		Matrix in [10]		Matrix in [11]	
		PSNR (dB)	MAE	PSNR (dB)	MAE	PSNR (dB)	MAE	PSNR (dB)	MAE	PSNR (dB)	MAE
256 × 256	0.2	22.6605	**13.4937**	**22.9093**	13.6029	19.3004	20.8691	19.8121	20.1697	19.8027	20.326
	0.4	28.0981	**6.5438**	**28.2923**	7.1719	25.0153	10.665	24.8638	11.1451	24.6087	11.6662
	0.6	**33.6027**	**3.3659**	33.3965	3.9245	28.9548	6.6186	29.112	6.7379	28.9423	7.1203
	0.8	**41.0839**	**1.3186**	40.4934	1.7148	33.4708	3.7215	34.381	3.5896	34.3176	3.8214
128 × 128	0.2	**23.131**	**12.5434**	23.0944	13.0996	20.2297	18.5022	19.8831	19.447	20.3421	18.573
	0.4	**28.9057**	**6.1362**	28.3261	6.9565	24.4056	10.9798	23.7888	12.1519	24.6381	11.2894
	0.6	33.5164	**3.3095**	**33.6502**	3.7189	29.4794	6.0557	28.8448	6.7576	28.8565	6.8646
	0.8	**41.0934**	**1.2557**	40.4846	1.6479	35.3713	2.955	33.8719	3.6074	33.8764	3.8433
64 × 64	0.2	22.5417	12.9205	**23.3979**	**11.8427**	19.7316	18.197	19.9574	18.5578	19.9684	18.4438
	0.4	**28.9637**	**5.6297**	28.8002	6.0466	25.0808	9.6379	24.2559	10.958	24.141	11.263
	0.6	33.1252	**3.1995**	**33.975**	3.2579	29.3844	5.5229	27.372	7.127	28.8159	6.5165
	0.8	**41.6295**	**1.1434**	39.3808	1.507	33.8811	2.8266	34.2642	3.1852	33.6178	3.6169
32 × 32	0.2	**21.9497**	**12.3345**	21.4686	13.4565	9.9053	64.1636	19.5569	17.6329	18.1682	21.2503
	0.4	**28.8353**	**5.3827**	27.4663	6.572	24.7045	8.6407	23.7865	10.4418	24.2994	10.2425
	0.6	**33.1936**	**2.9513**	32.205	3.5738	28.8559	5.0865	27.17	6.8196	28.885	5.802
	0.8	37.778	**1.2538**	**39.4997**	1.289	35.5054	2.1386	32.111	3.4847	33.7978	3.0413
16 × 16	0.2	**9.391**	**72.484**	8.1689	81.9014	8.8651	75.9757	9.1032	75.945	8.4494	82.0355
	0.4	**27.7557**	**5.3172**	26.5717	6.1481	10.1027	62.0664	22.5496	10.9147	22.3399	11.0919
	0.6	**31.8427**	**2.7285**	31.5169	3.1158	28.2701	4.366	30.3832	4.18	28.3195	5.2354
	0.8	**43.1952**	**0.8167**	40.5837	0.9692	35.1107	1.9557	35.4961	2.0199	32.6849	2.9363

## Data Availability

The data presented in this study are available on request from the corresponding author.
